# Three Out of Ten Working Patients Expect No Clinical Improvement of Their Ability to Perform Work-Related Knee-Demanding Activities After Total Knee Arthroplasty: A Multicenter Study

**DOI:** 10.1007/s10926-018-9823-5

**Published:** 2018-12-11

**Authors:** Yvonne van Zaanen, Rutger C. I. van Geenen, Thijs M. J. Pahlplatz, Arthur J. Kievit, Marco J. M. Hoozemans, Eric W. P. Bakker, Leendert Blankevoort, Matthias U. Schafroth, Daniel Haverkamp, Ton M. J. S. Vervest, Dirk H. P. W. Das, Walter van der Weegen, Vanessa A. Scholtes, Monique H. W. Frings-Dresen, P. Paul F. M. Kuijer

**Affiliations:** 1 Department Coronel Institute of Occupational Health, Amsterdam Public Health Research Institute, Amsterdam UMC, University of Amsterdam, Amsterdam, The Netherlands; 2 Department of Orthopaedics, Amphia Hospital, Breda, The Netherlands; 3 Orthopaedic Research Center Amsterdam, Amsterdam UMC, University of Amsterdam, Amsterdam, The Netherlands; 4 CORAL - Center for Orthopaedic Research Alkmaar, Department of Orthopaedics, Noordwest Ziekenhuisgroep, Alkmaar, The Netherlands; 5 Department of Human Movement Sciences, Faculty of Behavioural and Movement Sciences, Vrije Universiteit Amsterdam, Amsterdam Movement Sciences, Amsterdam, The Netherlands; 6 Master Evidence Based Practice, Amsterdam UMC, University of Amsterdam, Amsterdam, The Netherlands; 7 Slotervaart Center of Orthopedic Research and Education (SCORE), MC Slotervaart, Amsterdam, The Netherlands; 8 Department of Orthopaedic Surgery, Tergooi Hospital, Hilversum, The Netherlands; 9 Department and Research Center of Orthopaedic Surgery, St. Anna Hospital, Geldrop, The Netherlands; 10 Joint Research Orthopedic Surgery, OLVG+, Amsterdam, The Netherlands

**Keywords:** Knee arthroplasty, total, Vocational rehabilitation, Treatment outcome, Patient expectations, Work ability

## Abstract

*Purpose* Three out of ten patients do not return to work after total knee arthroplasty (TKA). Patient expectations are suggested to play a key role. What are patients’ expectations regarding the ability to perform work-related knee-demanding activities 6 months after TKA compared to their preoperative status? *Methods* A multi-center cross-sectional study was performed among 292 working patients listed for TKA. The Work Osteoarthritis or joint-Replacement Questionnaire (WORQ, range 0–100, minimal important difference 13) was used to assess the preoperatively experienced and expected ability to perform work-related knee-demanding activities 6 months postoperatively. Differences between the preoperative and expected WORQ scores were tested and the most difficult knee-demanding work-related activities were described. *Results* Two hundred thirty-six working patients (81%) completed the questionnaire. Patients’ expected WORQ score (Median = 75, IQR 60–86) was significantly (p < 0.01) higher than their preoperative WORQ score (Median = 44, IQR 35–56). A clinical improvement in ability to perform work-related knee-demanding activities was expected by 72% of the patients, while 28% of the patients expected no clinical improvement or even worse ability to perform work-related knee-demanding activities 6 months after TKA. Of the patients, 34% expected severe difficulty in kneeling, 30% in crouching and 17% in clambering 6 months after TKA. *Conclusions* Most patients have high expectations, especially regarding activities involving deep knee flexion. Remarkably, three out of ten patients expect no clinical improvement or even a worse ability to perform work-related knee-demanding activities 6 months postoperatively compared to their preoperative status. Therefore, addressing patients expectations seems useful in order to assure realistic expectations regarding work activities.

**Electronic supplementary material:**

The online version of this article (10.1007/s10926-018-9823-5) contains supplementary material, which is available to authorized users.

## Introduction

Participating in work is an important treatment goal for a growing number of patients after total knee arthroplasty (TKA) [1, 2]. The incidence of TKA is increasing in the Netherlands as well as in other Western countries [3–6]. This is also true in the working population, partly due to an increase in the age of retirement [7, 8] and partly due to TKA being performed more often at younger age [5, 9]. Surgeons have experienced difficulty in predicting functional outcomes also in the working TKA population [10]. Return to work (RTW) rates for this population vary between 68 and 85% [1, 11–13].

To ensure RTW for these patients, a better understanding of the barriers for RTW is needed. One of the barriers might be the ability to perform work-related knee-demanding activities such as kneeling, crouching and clambering, which remain difficult after TKA [11]. The ability to perform other work-related activities, such as operating foot pedals and standing, improve more after TKA [11]. Moreover, it is possible that RTW might be facilitated not only by the ability to perform work-related activities after TKA, but also whether this ability is in line with the patients’ preoperative expectations [14] and the corresponding measures they take. Current evidence suggests that, in general, patients with higher recovery expectations are more likely to RTW than patients with lower recovery expectations [15]. The relevance of patient expectations to functional outcomes after TKA is agreed upon, although not yet clearly understood [16–21]. The largest percentages of unfulfilled expectations concern kneeling (47%), squatting (44%) and walking middle long-distance (40%) [17], which are also important work-related activities. However, other important work-related activities [22] such as lifting, working below knee-height and walking on rough terrain, are not described. Patient expectations regarding these work-related knee-demanding activities still require investigation.

Since patients of working age expect to perform better on a variety of work-related knee-demanding activities after TKA, and often at demanding levels [2], we want to quantify patient expectations regarding ability to perform such work-related knee-demanding activities. This might not only serve to improve preoperative understanding and advice [17], but might also support timely postoperative guidance and integrated care for patients [1, 13, 23, 24].

Therefore the research questions are:

1. What are patients’ expectations regarding the ability to perform work-related knee-demanding activities 6 months after TKA compared to their preoperative status?
2. How many patients expect severe difficulty in the ability to perform work-related knee-demanding activities 6 months after TKA compared to their preoperative status?

## Patients and Methods

### Multi-center Study

A multi-center cross-sectional observational study was performed among patients listed for TKA at seven hospitals in the Netherlands. The hospitals were selected based on existing collaborations with Amsterdam University Medical Centers and were located in the northern, central and southern part of the Netherlands including city and rural areas and serve general TKA populations. Recruitment took place between June 24, 2014 and November 10, 2016. The study is followed by a prospective cohort study on functional recovery and RTW after TKA.

In the Netherlands work status is not systematically registered by orthopedic surgeons so all patients between 18 and 65 years listed for TKA were selected at each hospital. All these patients received a written invitation to participate from the researchers, including information about the study and an informed consent form. Informed consent was obtained from all individual participants included in the study. All patients who answered that they had a job and returned a signed informed consent form, before being operated, were included.

Preoperatively, the patients were asked to complete a questionnaire, either on paper or electronically, depending on their preference. A reminder to complete the questionnaire was sent after 2 weeks, up to two times, as long as they had not yet had their operation.

### Work Osteoarthritis or Joint-Replacement Questionnaire (WORQ) and Expectations

The patients’ preoperatively experienced difficulty in performing work-related knee-demanding activities and their expectations for 6 months postoperatively were assessed by the Work Osteoarthritis or joint-Replacement Questionnaire (WORQ, Online Appendix, Table 1) [22, 25]. The expectations of the patients were asked in the following manner: How much difficulty do you expect performing these activities at work, *6 months after TKA surgery*? The WORQ score consists of 13 items on work-related knee-demanding activities, like lifting and working below knee-height. These 13 activities are assessed on a 5-point scale, from 0 (extreme difficulty or unable to perform) to 4 (no difficulty at all), resulting in a converted total score between 0 (extreme difficulties) to 100 (no problems). A score of 71 or more is classified as being satisfied with their work ability with respect to the knee, while a score of 50 or less is considered as being unsatisfied [22]. The minimal clinically important difference (MID) of 13 points [22] was used to calculate how many patients expected a clinically relevant improvement in ability to perform work-related knee-demanding activities 6 months postoperatively. The WORQ scores were dichotomized to determine how many patients experienced severe difficulty with each of the 13 items. “Severe difficulty” and “extreme/unable to perform” were classified as “severe difficulty”. “Moderate,” “mild” and “no” difficulty were classified as “no severe difficulty.” This was done for both the preoperative scores and the expected scores for 6 months after TKA.

Patient characteristics are described, such as age, sex, educational level, BMI, and comorbidity. Three Knee Injury and Osteoarthritis Outcome Score (KOOS) subscales were used as an indication of disease severity, KOOS symptoms (7 items), KOOS pain (9 items) and KOOS Quality of Life (4 items). All KOOS subscales ranged from extreme problems (0) to no problems (100) and are validated [26, 27]. The job of the patient was categorized as knee-demanding if the patient reported to perform ‘often’ or ‘always’ on one or more of the following five activities crouching, kneeling, clambering, taking the stairs or lifting [28, 29]. Patient characteristics were also described for patients with and without a knee-demanding job.

We limited the possibility of selection bias by including seven hospitals providing TKA surgery for city and rural regions across the Netherlands. Selection bias due to a lack of computer skills was prevented by offering a paper copy of the questionnaire [30]. By asking the preoperatively experienced difficulty in knee-demanding activities and expectations for 6 months postoperatively in the same questionnaire we have tried to overcome response bias. By first asking about their present preoperatively perceived difficulty and next about their expected difficulty 6 months postoperatively, we expected to obtain more reliable and realistic expectations.

### Statistical Analysis

Descriptive statistics were used to describe the patient characteristics. Fisher-Freeman-Halton Exact test for categorical variables and Mann Whitney *U* test for continuous variables were used to test for statistical differences between patients with and without a knee demanding job.

For question 1, the preoperative and expected 6-months postoperative WORQ scores were calculated as well as the difference in scores. The Wilcoxon signed ranks test was used to assess the statistical difference between the paired preoperative and expected 6-months postoperative WORQ scores. For question 2, the percentages of patients were calculated with “severe difficulty” preoperatively and expected “severe difficulty” postoperatively in performing a knee-demanding activity. McNemar’s test for paired samples was used to test for a difference between the pre- and postoperative “severe difficulty” scores.

Only participants with no missing data or a maximum of one missing item on the 13 items of the WORQ scores were included for analysis. In case of one missing item this was imputed corresponding to similar items belonging to knee coordination or strenuous knee flexion activities [22]. Sensitivity analysis was performed for expected WORQ scores and expected differences in WORQ scores in patients with and without a knee demanding job. Statistical difference between groups was tested using Mann Whitney *U* test for continuous variables and Pearson Chi square test for dichotomous variables.

The IBM SPSS version 22 was used for all statistics (IBM, Armonk, NY, USA).

## Results

### Working TKA Patients—Characteristics

**Fig. 1:**
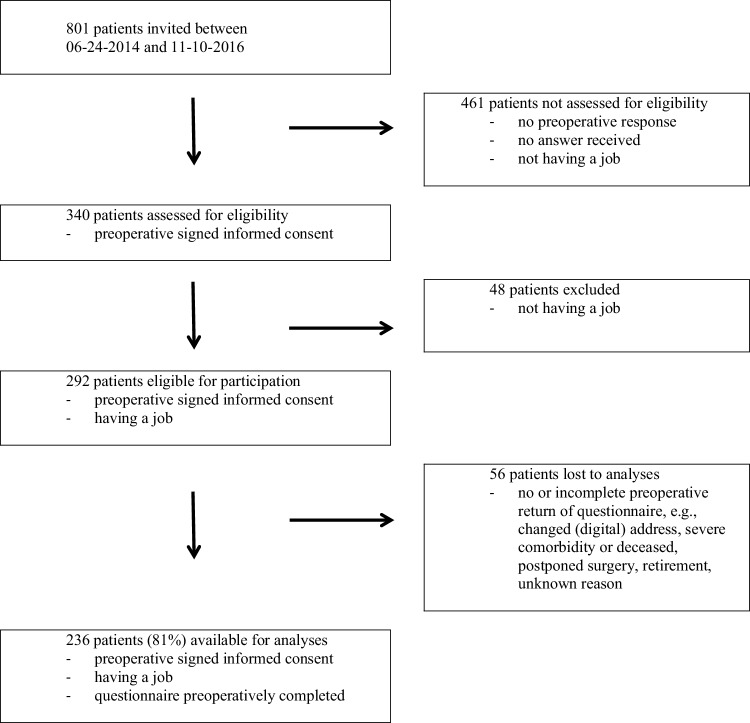
Flow chart of patient inclusion

Of 801 patients on the waiting list for TKA who received information about the study, 461 patients did not have a job, did not respond in time to participate before surgery or did not respond at all (58%). Three hundred and forty (n = 340) patients agreed to participate but 48 of these patients did not meet the inclusion criteria of having a job (paid or voluntary). Therefore, 292 patients were eligible to participate in the study: 36% of all patients contacted (Fig. 1). By November 10, 2016, 236 of these 292 patients returned their questionnaires, and these were used in the analyses: 81% of all eligible patients willing to participate.

The demographics of the study population are presented in Table 1. The median age at the time of completing the questionnaire was 59 years (IQR 54–62 years), while 104 participants (44%) were male and 132 (56%) female. The median Body Mass Index (BMI = body mass/body height2) was 29 (IQR 26–33) and 80% (N = 187) of the patients did not have another impairing disease. The median of the KOOS subscale for symptoms was 43 (IQR 32–57), the mean for pain was 38 (SD 17) and the median for Quality of Life was 25 (IQR 13–38). The questionnaire was completed by 134 patients less than 4 weeks before their TKA (57%); by 33 patients (14%), 4–8 weeks before; by 19 patients (8%), 8–12 weeks before; and by 24 patients (10%) more than 12 weeks preoperatively. For 26 patients (11%), the date of surgery was unknown. Of the study population, 95% (N = 224) desired to return to their current job after surgery, 12 patients (5%) did not, while 118 patients (50%) had a knee-demanding job.

**Table 1:** Preoperative characteristics of working patients < 65 years undergoing TKA

Variable		Mean (SD), median [IQR] or cases (percentage) n = 236	Knee-demanding job n = 118	No knee-demanding job n = 118
Age	Years	59 [54–62]	58 [53–61]^a^	60 [56–62]^a^
Sex	Male	104 (44%)	45 (38%)	59 (50%)
	Female	132 (56%)	73 (62%)	59 (50%)
Educational level	Lower vocational/general	51 (21%)	31 (26%)	20 (17%)
	Intermediate vocational/intermediate and higher general	128 (55%)	60 (51%)	68 (58%)
	Higher vocational/university	56 (24%)	26 (22%)	30 (25%)
BMI	Body mass/body height^2^ (kg/m^2^)	29 [26–33]	29 [26–32]	29 [26–33]
Comorbidity	No other impairing disease	187 (80%)	93 (79%)	94 (80%)
	One other impairing disease	34 (14%)	15 (13%)	19 (16%)
	More than one other impairing disease	14 (6%)	9 (8%)	5 (4%)
KOOS symptoms	0–100	43 [32–57]	43 [32–54]	43 [36–61]
KOOS pain	0-100	38 (17)	36 [21–47]^a^	42 [28–53]^a^
KOOS Quality of Life	0–100	25 [13–38]	19 [13–34]	25 [13–38]
Time before operation	< 4 weeks	134 (57%)	69 (59%)	65 (55%)
	4–8 weeks	33 (14%)	15 (13%)	18 (15%)
	8–12 weeks	19 (8%)	11 (9%)	8 (7%)
	> 12 weeks	24 (10%)	13 (11%)	11 (9%)
	Date of operation unknown	26 (11%)	10 (9%)	16 (14%)
Desire to return to current job	Yes	224 (95%)	112 (95%)	112 (95%)
	No	12 (5%)	6 (5%)	6 (5%)

On KOOS symptoms and KOOS pain two answers were missing, on educational level, BMI, comorbidity and KOOS Quality of Life one answer was missing (both < 1%). Due to integers not all percentages count up to 100

*KOOS* Knee Injury and Osteoarthritis Outcome Score

^a^Variable with a significant difference ≤ 0.05 between knee-demanding and no knee-demanding job, Fisher–Freeman–Halton exact test for categorical variables, Mann Whitney *U* test for continuous variables

### WORQ

**Fig. 2:**
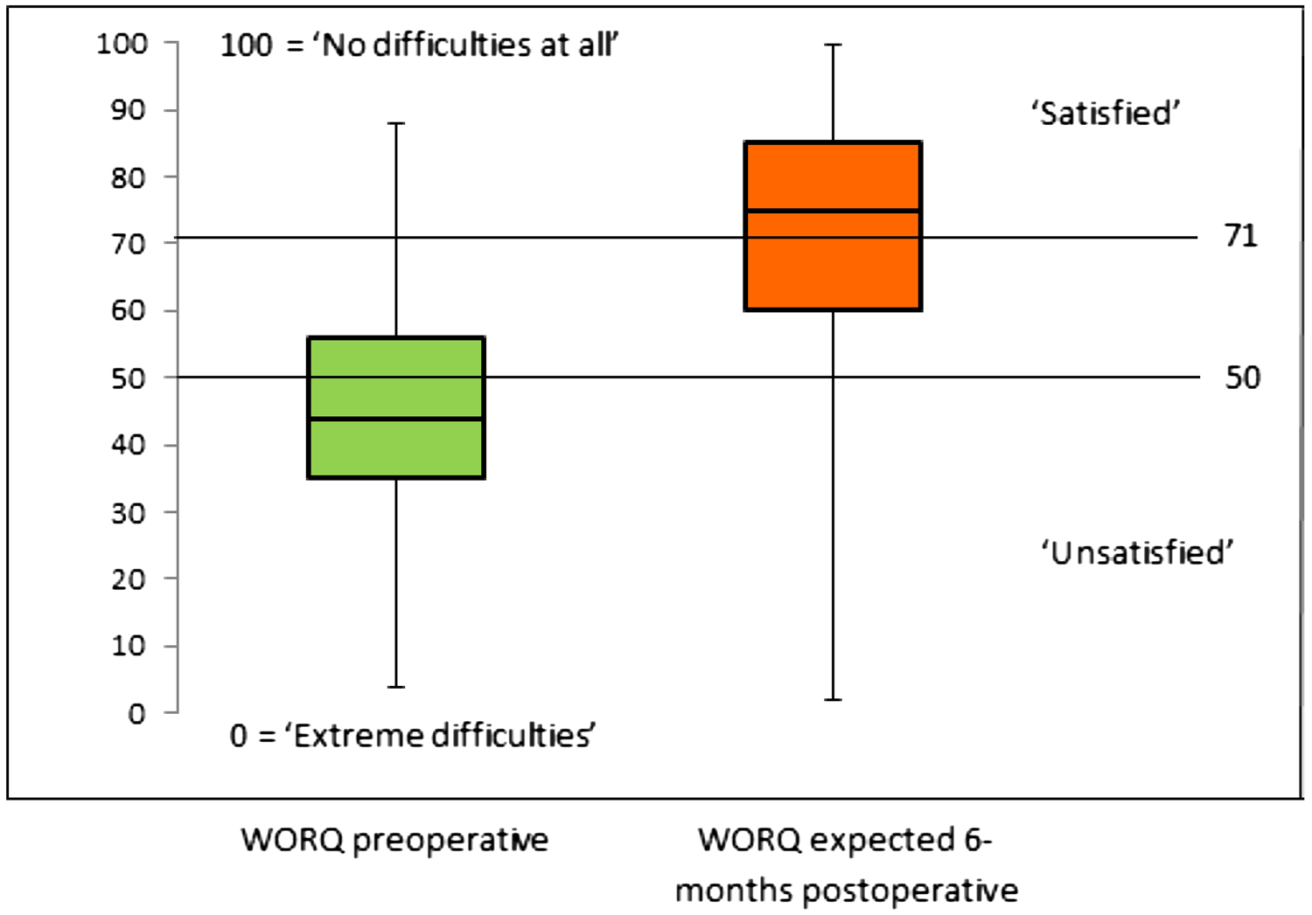
Boxplots of the experienced preoperative and the expected 6-months postoperative difficulties in ability to perform work-related knee-demanding activities measured by the WORQ (N = 236)

The median preoperative WORQ score was 44 [IQR 35–56]. In total, 66% of the patients (n = 156) reported a WORQ score of 50 or less, meaning an “unsatisfying” ability to perform work-related knee-demanding activities (Fig. 2). The median expected WORQ score 6 months after TKA was 75 [IQR 60–85] and significantly higher compared to the preoperative score (p < 0.01). 6 Months after TKA, 143 patients (61%) expected a score of 71 or higher, meaning a “satisfying” ability to perform knee-demanding activities, and 41 patients (17%) expected a WORQ score of 50 or less, meaning an “unsatisfying” ability. The median intra-individual change in scores was 29 [IQR 10–44, range − 37 to 90]. Of the patients, 72% (n = 171) expected a WORQ score of at least 13 points (MID) higher than their preoperative score, meaning that they expected a clinically important improvement. Of the patients who expected no clinically important improvement (N = 65, 28%), 32 patients (14%) expected no improvement or even a deterioration in performance. Only 7 WORQ-items were imputed of a total of 3068 items for the WORQ and the expected WORQ score (0.2%). Sensitivity analysis revealed no statistically significant difference between expected WORQ score and expected difference in WORQ score for patients with and without a knee demanding job (Table 2).

**Table 2:** WORQ score, Expected WORQ score and differences in expected scores among all working TKA patients and among TKA patients with and without a knee-demanding job

Variable	Working TKA patients n = 236 Median [IQR] or cases (percentage)	Knee-demanding job^a^ n = 118 Median [IQR] or cases (percentage)	No knee-demanding job n = 118 Median [IQR] or cases (percentage)	Difference between the groups of knee-demanding and no knee-demanding job
WORQ score	44 [IQR 35–56]	42 [IQR 33–54]	44 [IQR 37–56]	n.s.
Expected WORQ score	75 [IQR 60–85]	75 [IQR 59–85]	77 [IQR 60–87]	n.s.
Expected difference (WORQ-Expected WORQ)	29 [IQR 10–44]	29 [IQR 10–43]	33 [IQR 11–44]	n.s.
Expected WORQ score ≤ 50 (unsatisfied)	41 (17%)	23 (20%)	18 (15%)	n.s.
WORQ score ≥ 71 (satisfied)	143 (61%)	71 (60%)	72 (61%)	n.s.
MID (> 13 point)	171 (73%)	86 (73%)	85 (72%)	n.s.

*WORQ* Work Osteoarthritis or joint-Replacement Questionnaire

^a^The job was classified as knee demanding if the patient reported to perform ‘often’ or ‘always’ on one or more of the following five activities: crouching, kneeling, clambering, taking the stairs or lifting. No statistical differences (p > 0.05, n.s.) were found between knee-demanding and no knee-demanding job by Mann–Whitney *U* test for continuous variables, Pearson Chi-square test for dichotomous variables

### Severe Difficulty in Activities

**Fig. 3:**
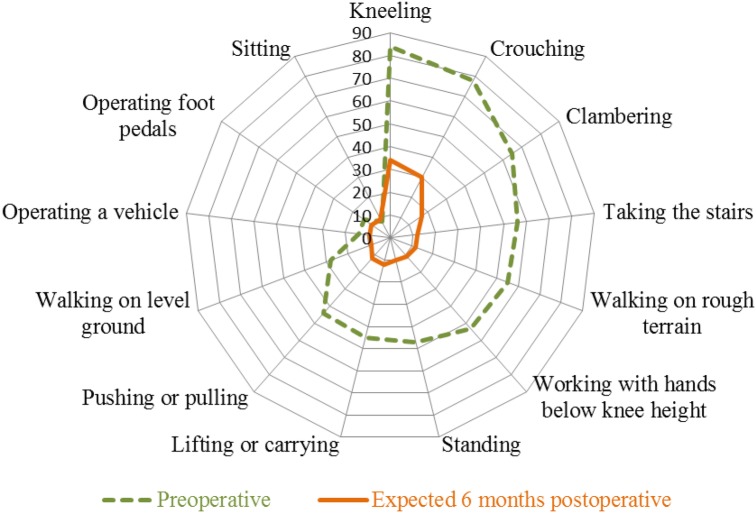
The percentage (%) of patients experiencing severe difficulty in their ability to perform work-related knee-demanding activities preoperatively and the percentage (%) of patients expecting severe difficulty 6 months postoperatively

Preoperatively, the largest percentage of patients experienced severe difficulty with kneeling (84%), followed by crouching (78%) and clambering (65%) (Fig. 3). The percentage of patients expecting severe difficulty 6 months postoperatively was largest for kneeling (34%), crouching (30%) and clambering (17%). For all other work-related knee-demanding activities, 9–12% of the patients expected severe difficulty 6 months postoperatively. Fifty percent (− Δ50%) fewer patients expected to have severe difficulty with kneeling 6 months postoperatively compared to preoperatively, and 48% fewer for crouching and clambering. The same was true for taking the stairs (− Δ44%), working with hands below knee height (− Δ42%), standing (− Δ37%), lifting or carrying (− Δ33%), pushing or pulling (− Δ33%) and walking on level ground (− Δ19%). For these nine activities, patients have significantly (p < 0.01) higher expectations regarding the ability to perform them after TKA compared to their preoperatively experienced ability. Only 5% fewer patients expected severe difficulty operating a vehicle (p = 0.07), 4% (p = 0.26) foot pedals and 1% (p = 0.86) sitting after TKA.

## Discussion

### WORQ

Patients have high expectations of TKA regarding their ability to perform work-related knee-demanding activities 6 months postoperatively. Their expected median WORQ score of 75 (IQR 60–86) was a little higher than the median WORQ score of 72 (IQR 54–83) 2 years after TKA, reported in a retrospective study among working-age Dutch TKA patients [11]. One out of four patients in our study expected a score above 86 and some even up to 100, with the latter meaning that they expect “no difficulties at all” in their ability to perform work-related knee-demanding activities 6 months postoperatively. In the above-mentioned retrospective study [11], 9% of the patients (n = 10) had a WORQ score of 100, evidence that it is achievable.

Of our study population, 72% expected an improvement in work-related knee-demanding activities that would be of clinical importance. Therefore, the majority of working patients expect that TKA will improve their ability to perform at work. This is in line with percentages found in studies on RTW after TKA, varying between 68 and 85% [1, 11–13]. Remarkably, 28% expected no clinical improvement and no less than 14% of all patients expected no improvement at all or even a worse performance 6 months after TKA. One explanation for this might be that these patients have low expectations because they expect a vocational rehabilitation period of more than 6 months.

In studies of RTW after TKA, percentages between 17% [12] and 30–40% [11, 31] have been found for no RTW within 6 months, also indicating a prolonged rehabilitation period. An expected prolonged period of recovery might especially be likely for patients performing knee-demanding work or patients receiving no vocational rehabilitation or integrated work-directed care. Other explanations might be that these patients expect improvement on other dimensions such as pain or symptoms, instead of improvement in ability to perform work-related knee-demanding activities. This is in line with the suggestion made by Haynes et al. [32] to inform patients (≤ 55 years) about not achieving substantially high levels of function after TKA, relative to their age and preoperative activity level.

If low expectations were influenced by having a knee-demanding job, we would have found differences in expected WORQ scores (Table 2). Instead of differences in outcome on WORQ scores, we found significant baseline differences for age and the subscale KOOS-pain (Table 1). We applied a sensitivity analyses for these baseline characteristics of the TKA patients by exploring the highest and lowest tertiles. Patients ≤ 55 year (n = 76, 32%) expected more improvement in WORQ score namely a median of 31 points [IQR 16–44] than patients ≥ 61 year (n = 88, 37%) who expected a median improvement of 23 points [IQR 8–38]. Patients reporting more pain (KOOS pain ≤ 29, n = 76, 32%) expected a median improvement in WORQ scores of 40 points [IQR 22–52] compared to 22 points [6–35] by patients reporting less pain (KOOS pain ≥ 46, n = 76, 32%). These findings highlight the importance of paying special attention to expected changes in the ability to perform work-related activities after TKA.

In all attempts to explain patients’ low expectations of their ability to perform work-related knee-demanding activities, it is important to explore whether these expectations are proven true 6 months after TKA, including the impact on work participation. While patients with higher recovery expectations are, in general, more likely to RTW [15] it seems important to determine what factors influence low expectations and what factors might contribute to guiding these patients toward the most achievable goals regarding the ability to perform work-related knee-demanding activities. The increasing number of working TKA patients in the upcoming decades, stresses the importance of managing low patient expectations in order to secure realistic expectations and hopefully thereby contributing to a timely and sustainable RTW of this patient group ‘at risk’.

### Severe Difficulty in Activities

Most patients (88–91%) expected no severe difficulties for 10 of the 13 work-related knee-demanding activities 6 months after TKA. However, patients did expect severe difficulty in kneeling (34%), crouching (30%) and clambering (17%) 6 months postoperatively. These expectations are in line with preoperative information regarding functional limitations after TKA [33]. However, most of the patients in our study expected more improvement in activities involving deep knee flexion; namely, kneeling, crouching and clambering, than in activities involving knee coordination, such as operating foot pedals and walking, according to the classification of Kievit et al. [22]. This contrasts with previous research [11] in which activities involving knee coordination improved most and activities involving deep knee flexion had improved least 2 years after TKA in working patients of comparable age (mean 60 years) and BMI (mean 29.5). Moreover, in this retrospective study 2 years after surgery, higher percentages of patients having severe difficulty in kneeling, crouching and clambering were found; namely, 48%, 45% and 25%, respectively. Impairments involving knee flexion following TKA have also been found in patients younger than 60 and were highlighted in a review on establishing realistic patient expectations [20, 34]. Tilbury et al. [17] found the greatest proportions of unfulfilled expectations in the same activities; namely, kneeling and squatting. Better insight into the effects of expectations and what is achievable with respect to performing work-related knee-demanding activities is needed to guide patients and health care professionals in setting high and achievable goals [16]. Based on our findings, it is possible that expectations about the ability to perform deep knee flexion activities are too high. For this, a follow-up study is required to assess what the actual WORQ outcomes are 6 months after surgery. The effectiveness of expectation modification in case of over-optimistic expectations for TKA surgery also requires more corresponding evidence [35].

Moreover, it seems important to assess whether setting achievable goals preoperatively [36] is helpful in attaining better individualized functional recovery and more satisfied patients postoperatively. Patients who have low expectations of their postoperative ability to perform knee demanding activities might thereby foresee a discrepancy between their ability and the required work demands. In that case, the possibilities of workplace adaptations to overcome their needs (adjustment latitude [37]) are of primary importance including support by their employer, co-workers and of course the occupational health experts involved. In general, patients with end-stage knee osteoarthritis are recommended to postpone surgery if possible by using effective non-operative treatment like losing body weight, exercising, and using pain medication to avoid complications due to surgery among other reasons [38, 39].

### Strengths and Limitations

Strengths of this study are the large sample size and high response rate of eligible patients willing to participate, the preoperative assessment of patient expectations and the specific data on expectations for work-related knee-demanding activities. Another strength is the inclusion of patients with and without knee demanding work which makes it possible to explore expectations for the distribution of knee demands at work. The classification of performing knee demanding activities at work was based on the self-reports of the patients whether their work involved ‘often’ or ‘always’ crouching, kneeling, clambering, taking the stairs or lifting, thereby taking into account established risk factors for knee osteoarthritis [29].

A limitation of our study is that we do not know why the contacted 461 patients did not participate. For instance, did they not have a job, did they not want to participate, or did they have too little time to fill in the questionnaire before surgery? Another limitation might be a selection bias for patients fluent in reading and writing, so we might have included less lowly educated patients than in the general working population. The educational level of our sample was lower than the general Dutch working population between 55 and 65 (11%, 61%, 28%, respectively) but in line with the working TKA population described in another Dutch multicenter study by Leichtenberg (low 29%, medium 45%, high 27%) [13, 40]. This might indicate that we included comparable numbers of low, medium and higher educated patients. The results of this multi-center study seem generalizable to Dutch working TKA patients registered in other hospitals in the Netherlands then the hospitals participating in this study, given the similarities in age, sex, educational level and disease severity [13].

Another limitation might concern the fact that the WORQ does not specify the exposure to work-related knee-demanding activities of each patient in terms of level, frequency or duration. However, asking about this exposure in more detail would probably not have resulted in more accurate answers, given findings that suggest that patients do not answer these types of questions reliably [41, 42]. We expect that the influence of a difference in time point while filling in the questionnaire before surgery is small because the majority of patients (71%) completed the questionnaire within 8 weeks before surgery and this is a relatively short time period compared to the long period that these knee osteoarthritis patients suffer from the complaints before being admitted to have surgery.

## Conclusions

In conclusion, seven out of ten patients have high expectations about their ability to perform work-related knee-demanding activities 6 months after TKA, especially regarding activities involving deep knee flexion. Remarkably, 28% of patients expected no clinical improvement, no improvement at all or even a deterioration of ability to perform work-related knee-demanding activities 6 months postoperatively compared to preoperatively. These results emphasize the importance of discussing these expectations pre- and postoperatively, so that health care professionals can timely advise patients on whether additional care is needed after TKA.

## Electronic supplementary material

Below is the link to the electronic supplementary material.

Supplementary material 1 (DOCX 13 KB)
