# Supplementary material for: Three Out of Ten Working Patients Expect No Clinical Improvement of Their Ability to Perform Work-Related Knee-Demanding Activities After Total Knee Arthroplasty: A Multicenter Study
Source: J Occup Rehabil. 2018 Dec 11;29(3):585–94. doi: 10.1007/s10926-018-9823-5 (PMC6675779; doi:10.1007/s10926-018-9823-5)
Supplement: Supplementary file 1 — Supplementary material 1 (DOCX 13 KB) [file 10926_2018_9823_MOESM1_ESM.docx]

**Appendix**

Table 1. Work Osteoarthritis or joint-Replacement Questionnaire (WORQ) [1]

| How much difficulty did you experience with the following activities during the last week because of your knee? | | | | | |
| --- | --- | --- | --- | --- | --- |
|  | None | Mild | Moderate | Severe | Extreme/Unable to perform |
| Crouching | □ | □ | □ | □ | □ |
| Kneeling | □ | □ | □ | □ | □ |
| Clambering | □ | □ | □ | □ | □ |
| Walking on level ground | □ | □ | □ | □ | □ |
| Operating a vehicle | □ | □ | □ | □ | □ |
| Operating foot pedals | □ | □ | □ | □ | □ |
| Sitting | □ | □ | □ | □ | □ |
| Walking on rough terrain | □ | □ | □ | □ | □ |
| Taking the stairs | □ | □ | □ | □ | □ |
| Standing | □ | □ | □ | □ | □ |
| Lifting or carrying | □ | □ | □ | □ | □ |
| Pushing or pulling | □ | □ | □ | □ | □ |
| Working with hands below knee height | □ | □ | □ | □ | □ |

1. Kievit, A. J., Kuijer, P. P., Kievit, R. A., Sierevelt, I. N., Blankevoort, L., & Frings-Dresen, M. H. (2014). A reliable, valid and responsive questionnaire to score the impact of knee complaints on work following total knee arthroplasty: the WORQ. *J Arthroplasty, 29*(6), 1169-1175 e1162, doi:10.1016/j.arth.2014.01.016.
